# Biopsy vs comprehensive embryo/blastocyst analysis: a closer look at embryonic chromosome evaluation

**DOI:** 10.1093/hropen/hoaf013

**Published:** 2025-03-12

**Authors:** Jian Xu, Zhiheng Chen, Meiyi Li, Ling Sun

**Affiliations:** Center of Reproductive Medicine, Guangzhou Women and Children’s Medical Center, Guangzhou Medical University, Guangzhou, Guangdong Province, China; Center of Reproductive Medicine, Guangzhou Women and Children’s Medical Center, Guangzhou Medical University, Guangzhou, Guangdong Province, China; Center of Reproductive Medicine, Guangzhou Women and Children’s Medical Center, Guangzhou Medical University, Guangzhou, Guangdong Province, China; Center of Reproductive Medicine, Guangzhou Women and Children’s Medical Center, Guangzhou Medical University, Guangzhou, Guangdong Province, China

**Keywords:** whole embryo, trophectoderm biopsy, cytogenetic constitution, mosaic aneuploidy, pre-implantation embryos, human

## Abstract

**STUDY QUESTION:**

Compared with embryonic cytogenetic constitution of biopsied samples in human pre-implantation embryos, are there any differences in whole embryos?

**SUMMARY ANSWER:**

Whole embryos exhibit a significantly higher euploidy rate and reduction in mosaic aneuploidy rate compared to biopsied samples.

**WHAT IS KNOWN ALREADY:**

Much of the existing evidence of cytogenetic constitution of human pre-implantation embryos is based on biopsied cells obtained from blastomeres or trophectoderm biopsies. The mosaic rate of biopsies taken from blastocyst trophectoderm ranges widely, from 2% to 25%.

**STUDY DESIGN, SIZE, DURATION:**

We investigated the embryonic cytogenetic constitution of 221 whole human embryos/blastocysts from 2019 to 2022, including 41 high-quality blastocysts, 57 low-quality blastocysts, and 123 arrested embryos/blastocysts.

**PARTICIPANTS/MATERIALS, SETTING, METHODS:**

The cytogenetic constitution of whole embryos/blastocysts was assessed using next-generation sequencing. Mosaicism was diagnosed using a cut-off threshold of 30–70%, with embryos displaying 30–70% aneuploid cells classified as mosaic.

**MAIN RESULTS AND THE ROLE OF CHANCE:**

Among high-quality blastocysts, the euploidy rate was 82.9%, with a remarkably low mosaic aneuploidy of only 2.5%. The euploidy rates of viable low-quality blastocysts and arrested embryos/blastocysts were 38.6% and 13.0%, respectively. The mosaic aneuploidy rate decreased progressively with embryonic development, from 93.2% at the cleavage stage to 40% at the blastocyst stage. Chaotic aneuploidy was the primary factor (66.1%, 39/59) contributing to embryonic arrest at the cleavage stage. Additionally, 26.1% of embryos/blastocysts exhibited segmental aneuploidy, with segmental duplications (30.6%, 22/72) and deletions (54.2%, 39/72) being the most common types of segmental aneuploidy.

**LIMITATIONS, REASONS FOR CAUTION:**

The sample size in this study is relatively small, especially in the subgroup analysis. Furthermore, whole-embryo analysis is not a foolproof diagnostic method, since it may underestimate the presence of mosaicism.

**WIDER IMPLICATIONS OF THE FINDINGS:**

The cytogenetic constitution of whole embryos provides a more accurate reflection of their physiological state compared to biopsied samples. The low mosaic aneuploidy rate in high-quality blastocysts supports the practice of transferring mosaic embryos in patients without euploid embryos. If blastocysts reach stage III by Day 6 post-fertilization, nearly half are euploid, suggesting that extending embryo culture to Day 7 may be beneficial in cases where high-quality embryos are lacking.

**STUDY FUNDING/COMPETING INTEREST(S):**

This study was supported by the Natural Science Foundation of Guangdong Province (No. 2023A1515010250) and Pilot Program-China Reproductive Health Public Welfare Fund Project (No. SZ202413). The authors report no conflicts of interest.

**TRIAL REGISTRATION NUMBER:**

N/A.

WHAT DOES THIS MEAN FOR PATIENTS? Chromosomal normality plays an important role in determining the success of embryo transfer. However, much of the evidence is derived from biopsied cells, which might not fully represent the condition of the whole embryo.

In this study, we investigated the chromosomal makeup of whole embryos at different stages before implantation. Our results showed that many embryos which, based on biopsy results, were thought to be made up of a mixture of cells including many with abnormal chromosomal content, are actually normal when examined as whole embryos. This suggests that these embryos may still have the potential for a successful pregnancy.

In addition, some embryos grow slowly in the IVF laboratory, taking 6 days to reach the level of development called blastocyst stage III. We found that half of these embryos had normal chromosomes, suggesting that extending the culture of these embryos to Day 7 could be helpful, especially when there are no high-quality embryos.

Furthermore, a common concern among patients is the cause of arrest in the development of the embryo. Our analysis showed that chromosomal abnormalities, involving multiple chromosomes, are the main reason for the arrest of embryo development at the cleavage stage (when the large fertilized egg is dividing into many smaller cells).

## Introduction

The euploidy status of pre-implantation embryos is very important for successful embryo transfer and subsequent live birth outcome, making it a central focus of embryological research.

Aneuploidy in embryos can result from misallocation of chromosomes during meiosis, either in the egg or sperm, or from mitotic errors post-fertilization (McCoy *et al.*, 2015; Lee and Kiessling, 2017). It is important to note that much of the current understanding of aneuploidy originates from biopsied cells obtained from blastomeres or trophectoderm (TE) biopsies (McCoy *et al.*, 2015; Shahbazi *et al.*, 2020). Due to the lack of cell cycle checkpoints (Lee and Kiessling, 2017) in embryos prior to zygotic genome activation, which occurs around the four- to eight-cell stage (Braude *et al.*, 1988), early human embryos exhibit a high rate of mitotic errors, particularly in the first two to three mitotic divisions (Mantikou *et al.*, 2012). These mitotic errors often result in mosaic embryos (Fragouli *et al.*, 2019), consisting of cells with two or more different chromosomal complements, either normal and abnormal, or different types of abnormal cells (Jacobs *et al.*, 2017). It has been reported that up to 73% of cleavage-stage embryos exhibit mosaicism (Mantikou *et al.*, 2012). At the blastocyst stage, the mosaic rate of biopsies taken from TE ranges widely, from 2% to 25% (Popovic *et al.*, 2020, 2024; Armstrong *et al.*, 2023; Rana *et al.*, 2023; De Witte *et al.*, 2024). These results differ substantially from studies of prenatal diagnosis, which have reported placental mosaicism rate of <2% and an estimated live birth mosaicism rates below 0.2% (Popovic *et al.*, 2020).

This inconsistency has sparked an ongoing debate as to whether the detected mosaicism accurately reflects the true incidence of mosaicism or if it is a result of technical artifacts (Popovic *et al.*, 2020; Marin *et al.*, 2021). Consequently, several studies based on biopsy data have highlighted the limitation that biopsied cells may not represent the entire embryo, and it is necessary to study the whole embryo to verify the conclusions reached (Babariya *et al.*, 2017). Therefore, the exploration of data acquired from whole embryos holds significant clinical implications. However, these embryos, which are suitable for biopsy, are typically reserved for clinical use, making their availability for research purposes impractical. As a result, there is a lack of studies investigating the genomic status of whole embryos in high-quality blastocysts.

Furthermore, low-quality blastocysts, another subgroup of pre-implantation embryos, have often been overlooked in previous studies. Current research focuses on two categories: embryos that meet the biopsy criteria, and arrested embryos that failed to develop for at least 24 h in the embryo culture system. However, in actual clinical practice, there are still embryos that exhibit slightly delayed growth, falling outside the consensus criteria. For instance, Day 6 (D6) embryos, which do not meet the biopsy criteria, but can continue to develop. Investigating the cytogenetic characteristics of these embryos is also of substantial clinical relevance.

To address these limitations, we conducted a study using embryos generously donated by patients. In this study, we aimed to include embryos at all possible stages of pre-implantation development, including high-quality blastocysts suitable for biopsy or transfer, delayed D6 blastocysts, as well as compact- (CP) and cleavage-stage embryos that failed to form blastocysts by the end of 6 days post-fertilization (dpf). To ensure the reliability and statistical validity of the study, we meticulously chose a large sample size of 221 embryos, encompassing all the developmental stages. Moreover, given the enhanced capacity of next-generation sequencing (NGS) in detecting mosaic abnormalities in embryos, we opted to utilize this method for comprehensive screening the entire embryo/blastocyst (Fragouli *et al.*, 2019).

## Materials and methods

### Ethical approval

This study received ethical approval from the Reproductive Medical Ethics Committee of Guangzhou Women and Children’s Medical Center (2023-200A01). All embryos used in this study were donated with the written informed consent of the patients.

### Source of embryos

The embryos utilized in this study originated from two distinct sources.

**Figure 1.**
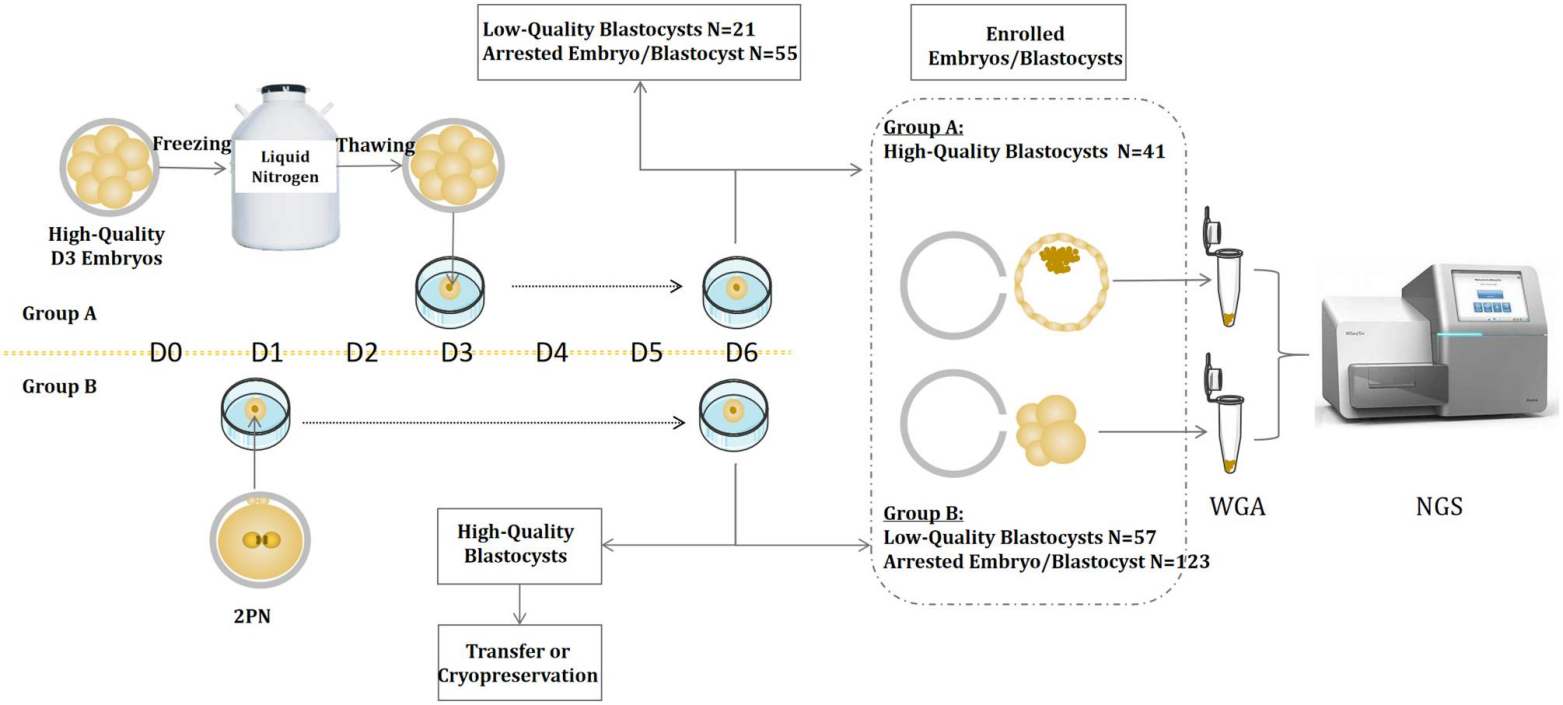
**Experimental design of the study**. The embryos used in this study originated from two distinct sources. Group A: 117 high-quality embryos at cleavage stage were thawed and cultured in sequential media until reaching 6 days post-fertilization (dpf). Group B: Embryos from 33 patients were cultured to 6 dpf, with high-quality blastocysts either being transferred or cryopreserved. One hundred and eighty low-quality blastocysts or embryos exhibiting developmental arrest were donated for this research. On the 6th dpf, the zona pellucida was removed from all embryos. Cells derived from the remaining intact cleavage-stage embryos or blastocysts were gathered as a singular sample. Whole-genome amplification (WGA) was performed to ensure sufficient DNA yield, and the cytogenetic constitution of the embryos was evaluated using next-generation sequencing (NGS).

In Group A, a total of 117 embryos were obtained from 15 couples who had previously achieved at least one live birth through a prior IVF cycle. The maternal ages of the participants ranged from 24 to 37 years, with a mean age of 30.9 years. These embryos exhibited characteristics indicating good quality, including 2–5 blastomeres on 2 dpf, 6–10 blastomeres on 3 dpf, less than 20% fragmentation, and equal-sized blastomeres (Vanneste *et al.*, 2009). Following cryopreservation through vitrification in accordance with the manufacturer’s protocol (ARSCI Inc., Longueuil, Canada), the embryos were stored in a liquid nitrogen storage facility. Couples generously donated these embryos after delivery, from May 2021 to September 2021. Subsequently, 117 embryos were thawed and cultured in sequential G1/G2 media (Vitrolife, Frolunda, Sweden) until reaching 6 dpf (Fig. 1).

A cohort of 33 patients, with an average maternal age of 33.3 years, was included in group B. The standard embryo culture workflow was followed, in which oocytes were fertilized using conventional IVF or ICSI. Fertilization was assessed 16–18 h after insemination. Normal fertilized zygotes were cultured individually in sequential G1/G2 media (Vitrolife) until reaching 6 dpf. The culture was conducted under controlled conditions of 6% CO_2_, 5% O_2_, and 37°C. High-quality blastocysts were either transferred or cryopreserved. Finally, a total of 180 low-quality blastocysts or embryos with developmental arrest were graciously donated for this study (Fig. 1).

### Grading of cleavage embryos and blastocysts

Cleavage-stage embryos were scored according to the Istanbul and Vienna consensus (Alpha Scientists in Reproductive Medicine and ESHRE Special Interest Group of Embryology, 2011; ESHRE Special Interest Group of Embryology and Alpha Scientists in Reproductive Medicine, 2017). Similarly, blastocyst quality was evaluated utilizing the grading criteria outlined by Gardner and Schoolcraft (Gardner *et al.*, 2000).

### Definition of developmental arrest, viable low-quality blastocyst, and biopsiable blastocyst

Developmental arrest embryo/blastocyst is defined as the absence of cleaving activity and failure to demonstrate any signs of mitotic cellular division for at least 24 h (Alpha Scientists in Reproductive Medicine and ESHRE Special Interest Group of Embryology, 2011). It also includes embryos that remain in the CP stage at 6 dpf and failed to progress to the blastocyst stage. Viable low-quality blastocysts were defined as blastocysts that exhibit cleaving activity within 24 h but are not suitable for transfer, biopsy, or cryopreservation on 6 dpf (Hanson *et al.*, 2021). These embryos are routinely discarded in clinical practice (Hammond *et al.*, 2018). Blastocysts with a 5/6 dpf score ≥4CB/4BC were classified as high-quality and suitable for transfer, biopsy, or cryopreservation and were defined as biopsiable blastocysts in this study (Yu *et al.*, 2022).

### Zona pellucida removal and whole-genome amplification

At 6 dpf, all embryos included in the study underwent meticulous preparation for genetic analysis. The zona pellucida was gently removed from embryos, and cells derived from the remaining intact cleavage-stage embryos or blastocysts were collected as a single specimen. Multiple displacement amplification (MDA, Qiagen, Hilden, Germany) was employed for whole-genome amplification (WGA) to yield an ample quantity of DNA suitable for comprehensive analysis. The MDA reactions were incubated at a temperature of 30°C for a duration of 8 h and subsequently subjected to heat inactivation at 65°C for a brief span of 3 min, in accordance with the protocol outlined by the manufacturer (Ou *et al.*, 2020).

### Next-generation sequencing

The Illumina MiSeq platform (Illumina, San Diego, CA, USA) was employed for NGS. Approximately 1.5 million fragments of amplified DNA from each individual sample were subjected to sequencing. The chromosomal copy number variants (CNVs) were analyzed using the PGXcloud cloud server (available at http://www.pgxcloud.com/, Jabrehoo, Beijing, China) (Ou *et al.*, 2020). All profile reports underwent independent analysis by two skilled laboratory technicians. In the event of any discrepancies in the final assessment between these technicians, a consensus was achieved through further deliberation among the team members.

### Euploidy analysis of embryo/blastocyst

Embryos were classified as euploid, aneuploid, or mosaic. Mosaic diagnosis utilized cut-off levels of 30% and 70%. Embryos with less than 30% aneuploid cells were deemed euploid, while those with 30–70% aneuploid cells were considered mosaic, and those with over 70% aneuploid cells were classified as aneuploidy (De Rycke *et al.*, 2022). Segmental aneuploidy was identified when a chromosome fragment larger than 5 Mb deviated from the standard thresholds for euploidy (Escribà *et al.*, 2019). Chaotic mosaicism, commonly observed in cleavage-stage embryos, is characterized by the presence of more than three chromosomal complements in each cell (Cavazza *et al.*, 2021). Blastocysts were categorized as ‘no-result’ in cases of amplification failure, signifying insufficient genetic material for analysis. Additionally, blastocysts were also classified as ‘no-result’ if results were non-concurrent, indicating wide scatter in data and failure to meet quality control standards (Neal *et al.*, 2019). In our study, the chromosomal copy numbers (CNs) were assumed to be diploid. Specific CNs were calculated as the mean of the replicates, while concordances were determined based on their SD. A concordance SD ≥3.5 was considered non-concurrent and unreliable for diagnosis.

## Results

### Embryo distribution across developmental stages

In Group A, 117 high-quality embryos on Day 3 were cultured until 6 dpf. Morphological analysis identified 41 blastocysts of high quality, suitable for biopsy or transfer. Additionally, 21 blastocysts were classified as viable but low quality, while 55 embryos were arrested at various developmental stages.

In Group B, all viable blastocysts of high quality, suitable for transfer or biopsy, were utilized for clinical purposes. As a result, only low-quality blastocysts and arrested embryos/blastocysts, totaling 180 embryos, remained in this group. Among these, 57 embryos were classified as viable but low quality, consisting of 13 blastocysts at stages I–II and 44 blastocysts at stages III–V. Additionally, 123 embryos were arrested at various stages of development, including 75 at the cleavage stage, 25 at the CP stage, 13 at stages I–II of blastocyst development, and 10 at stages III–V of blastocyst development (Table 1).

**Table 1.** The distribution of embryos among different developmental stages and chromosomal copy number variants of enrolled embryos.

	Group A				Group B			
		NGS test				NGS test		
	No. embryos	No results	Normal CNV	Abnormal CNV	No. embryos	No results	Normal CNV	Abnormal CNV
**Biopsiable blastocyst**	**41**	**0**	**34**	**7**	**0**	0	0	0
**Viable low-quality blastocyst**	**21**				**57**	**0**	**22**	**35**
I–II stage blastocysts	8				13	0	2	11
III–V stage blastocysts	13				44	0	20	24
**Developmental arrest**	**55**				**123**	**10**	**16**	**97**
Cleavage stage arrest	20				75	8	8	59
CP-stage arrest	17				25	1	3	21
I–II stage blastocysts	18				13	1	0	12
III–V stage blastocysts	0				10	0	5	5
**Total embryos**	**117**				**180**	**10**	**38**	**132**

Group A: 117 high-quality embryos at cleavage stage were thawed and cultured in sequential media until reaching 6 days post-fertilization (dpf). Group B: Embryos from 33 patients were cultured to 6 dpf, with high-quality blastocysts either being transferred or cryopreserved, leaving 57 viable low-quality blastocysts and 123 arrested embryos. NGS: next-generation sequencing; CNV: copy number variants; CP: compact.

### Euploidy rate and categories of chromosomal aberrations

A total of 221 embryos were included in the euploid analysis. Among these, 41 biopsiable embryos originated from Group A, while 57 low-quality blastocysts and 123 arrested embryos were sourced from Group B. It is worth noting that 10 embryos failed to yield results due to data scattering or failure to meet quality control standards; all of them were from arrested embryos (Table 1).

**Figure 2.**
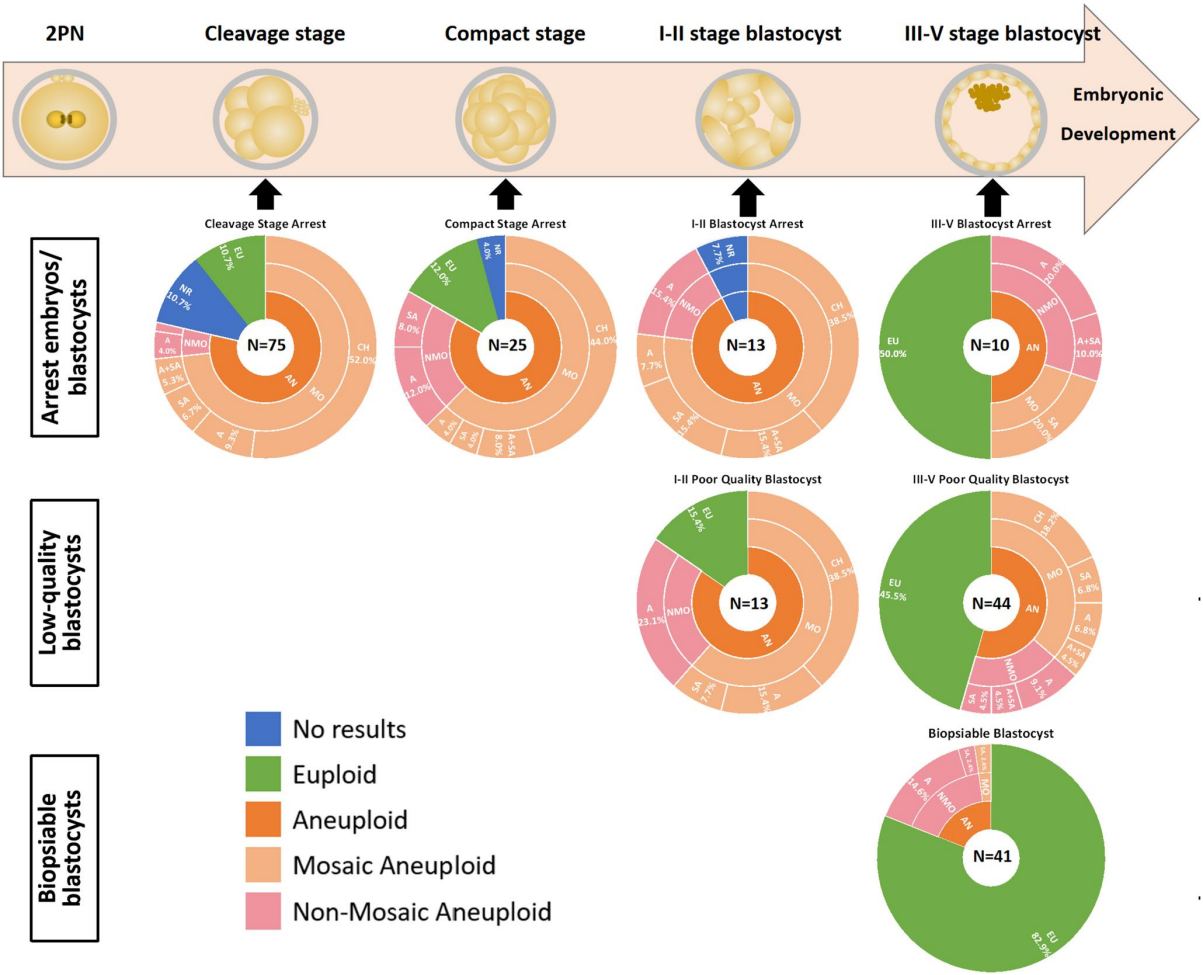
**Distribution of cytogenetic constitution in human preimplantation embryos/blastocysts**. Of the embryos analyzed, 10 failed to produce any results, all of which were categorized within the embryonic arrest group. Analyzing the remaining 211 informative embryos, it was found that the euploidy rate exceeded 80% in high-quality blastocysts. For viable low-quality blastocysts, nearly half of those reaching stage III or above were classified as euploid. The proportion of chromosomal mosaic aneuploidy decreased as the blastocyst developmental stage advanced. In the case of arrested embryos/blastocysts, it was observed that with the development of the embryo, the euploidy rate increased, and the mosaic aneuploidy rate decreased. It was also noted that 90% of cleavage-stage embryonic arrest was due to chaotic aneuploidy. NR: no results; EU: euploidy; AN: aneuploidy; MO: mosaic aneuploidy; NMO: non-mosaic aneuploidy; A: whole chromosomal aneuploidy; SA: segmental aneuploidy; CH: chaotic aneuploidy.

Analyzing the remaining 211 informative embryos, it was found that among the 41 high-quality blastocysts from group A, there was a notably high euploidy rate of 82.9% (34/41) (Table 1). Only seven blastocysts exhibited abnormal CNVs, characterized by single whole chromosome abnormalities (12.2%, 5/41), or single segmental abnormalities (4.9%, 2/41). The rate of mosaic aneuploidy was minimal, accounting for only 2.5% (1/41) (Supplementary Table S1, Fig. 2).

Among the 57 viable low-quality blastocysts, 38.6% (22/57) were euploid. However, additional analysis based on blastocyst stages demonstrated variations in the euploidy rates. For stages I–II, the euploidy rate was 15.4% (2/13) (Table 1), with mosaic aneuploidy being the predominant type, accounting for 72.7% (8/11) (Supplementary Table S1, Fig. 2). In contrast, the euploidy rate for blastocysts at stage III or above was 45.5% (20/44) (Table 1), with mosaic aneuploidy declining to 66.7% (16/24) (Supplementary Table S1, Fig. 2).

Out of the 123 embryos that were arrested at various developmental stages, 10 failed to yield any results. The overall euploidy rate was 13.0% (16/123), with variation across developmental stages (Table 1).

Among the 123 arrested embryos, 75 were arrested at the cleavage stage. Eight of these embryos had been arrested for more than 48 h and exhibited significant differences in size and fragmentation; nevertheless, they were still classified as euploid. The euploidy rate for this subgroup was 10.7% (8/75). Additionally, 25 embryos were arrested at the CP stage, with 3 classified as euploid, yielding a euploidy rate of 12.0% (3/25). Thirteen embryos arrested at stages I–II of blastocyst development were all found to be aneuploid. There were 10 cases that were arrested at stage III or above, with a euploidy rate of 50% (5/10) (Table 1).

The types of aneuploidies in the arrested group were similar to those observed in the low-quality viable blastocyst group. The rate of mosaic aneuploidy gradually decreased with embryonic development, from 93.2% (55/59) at the cleavage stage to 40% (2 out of 5) at blastocyst over stage III. Similarly, chaotic aneuploidy decreased from 66.1% (39/59) in arrested cleavage-stage embryos to 52.4% (11/21) in CP stage arrested embryos, and further to 41.7% (5/12) in I–II blastocyst stages arrested embryos (Supplementary Table S1, Fig. 2).

### Characteristics of chromosomal aberrations

The aberration pattern of segmental aneuploidy differs slightly from that of whole chromosome aneuploidy. Whole chromosome aneuploidy can involve the gain or loss of single or double whole chromosomes. In contrast, segmental aneuploidy involves the gain or loss of one or two chromosomal segments (Supplementary Table S1, Fig. 2).

**Figure 3.**
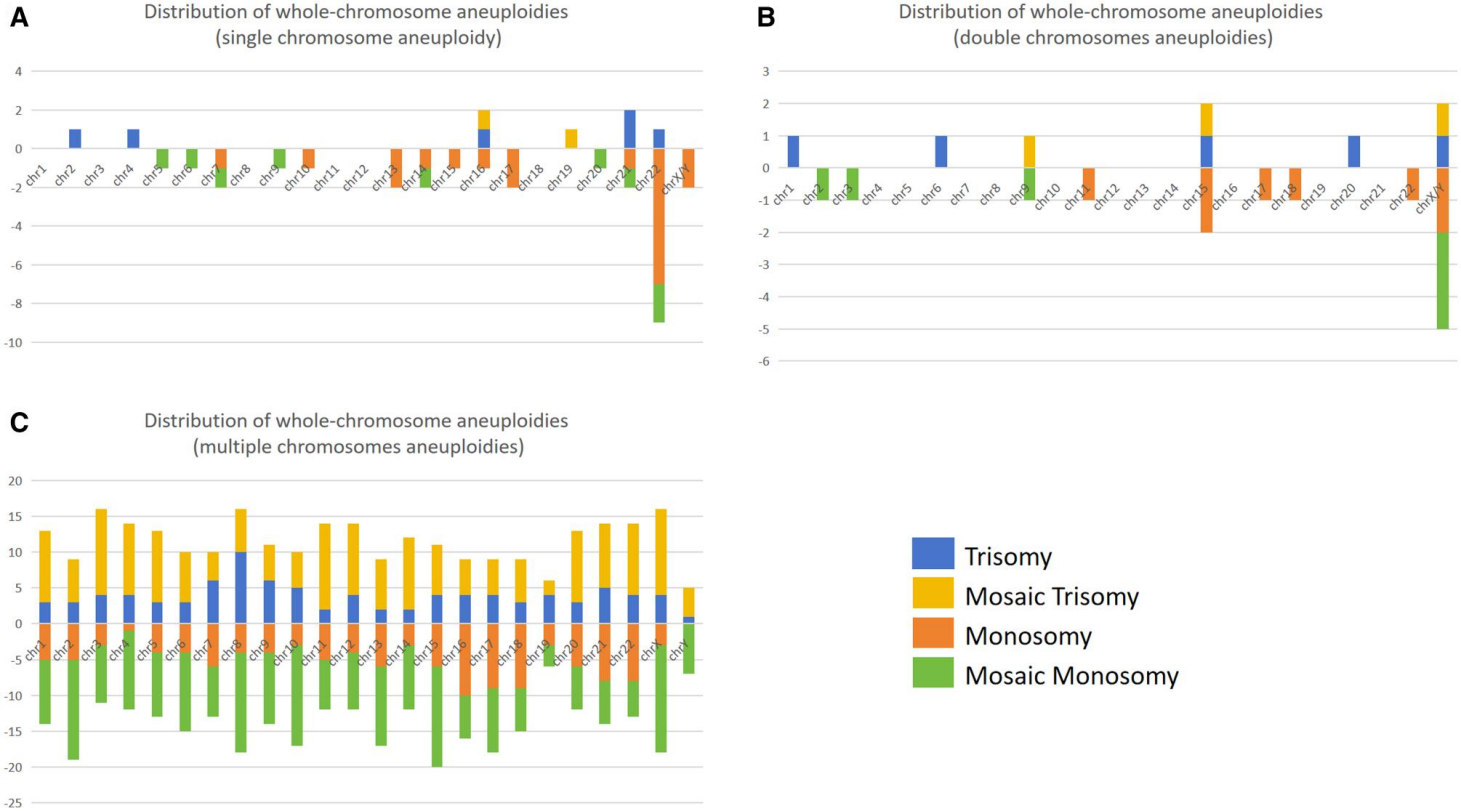
**Characteristics of whole chromosome deletion or duplication**. The horizontal axis represents different chromosomes, while the vertical axes indicate the frequency of chromosome gain (upward) and chromosome loss (downward), respectively. (**A**) Distribution of erroneous chromosomes in single whole-chromosome aneuploidy embryos, mainly involving chromosome 22 trisomy or monosomy. (**B**) Distribution of erroneous chromosomes in double chromosomes aneuploidy embryos, predominantly involving sex chromosome. (**C**) The erroneous chromosomes are randomly distributed in multiple chromosome aneuploidy embryos.

Single whole-chromosome aneuploidy was found in 36 embryos, characterized by the main features of gain or loss of chromosome 22 (Fig. 3A). Double whole-chromosome aneuploidies were identified in 11 embryos, with errors predominantly occurring in the sex chromosomes (Fig. 3B). Additionally, 68 embryos exhibited three or more whole-chromosome aneuploidies, termed chaotic aneuploidies, resulting in a random distribution of chromosomes (Fig. 3C).

**Figure 4.**
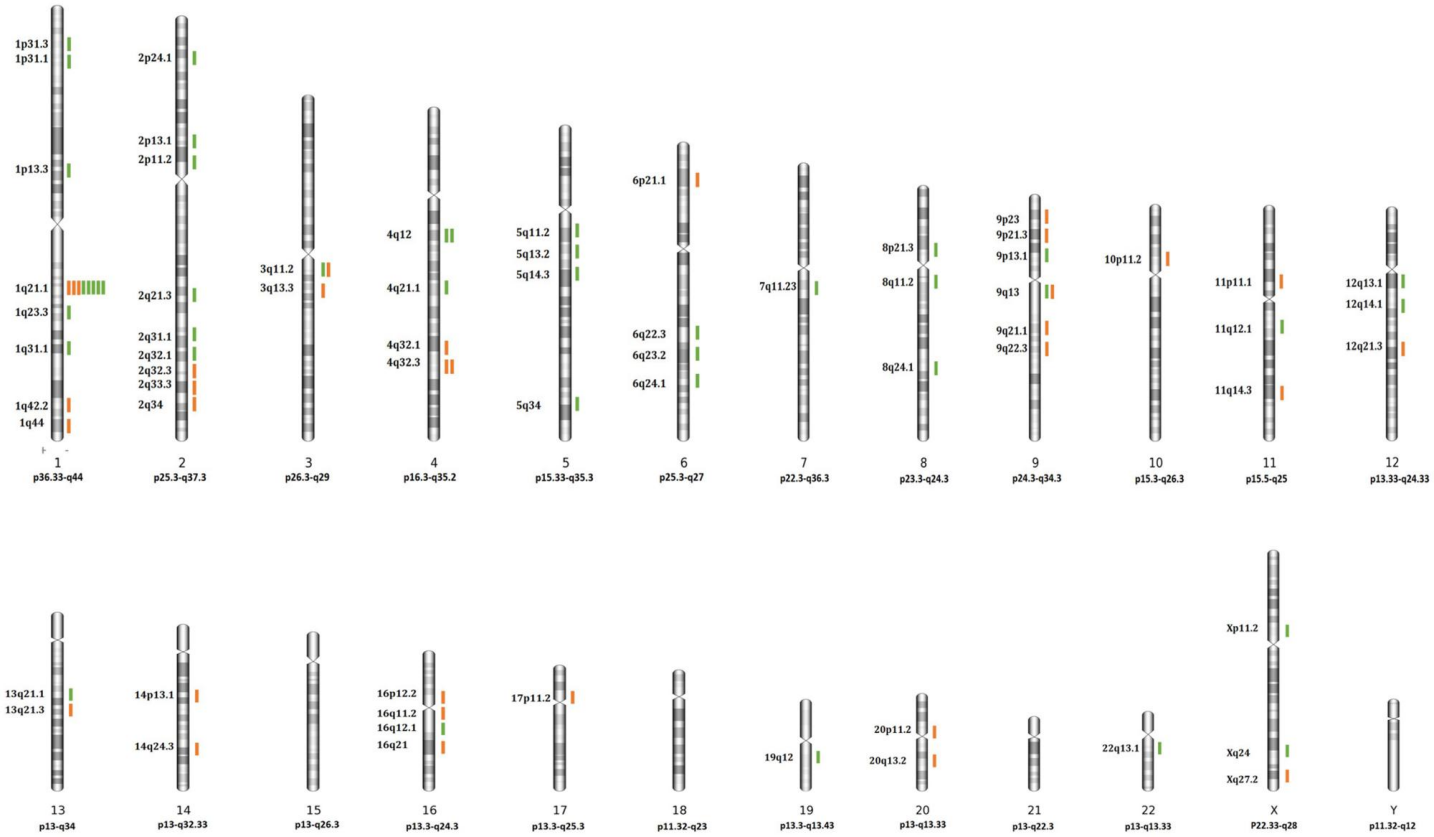
**Locations of chromosomal breakage sites**. Observed sites and frequency of chromosomal breakage in pre-implantation embryos/blastocysts are shown in the chromosomal ideogram, including mosaic (green lines), and non-mosaic breakpoints (brown lines). The 1q21.1 region was identified as the unique hot point of chromosomal breakage.

Out of the 211 informative embryos/blastocysts, 55 (26.1%) exhibited segmental aneuploidy. Segmental aneuploidy can occur independently or in conjunction with whole chromosome deletions or duplications. However, unlike whole chromosome gain or loss, embryos with segmental aneuploidies typically exhibit only one to two breakpoints (90.9%, 50/55) regardless of the developmental stage. In our study, a total of 75 breakpoints were identified, with most appearing randomly, aside from a breakpoint located at 1q21. Mosaic segmental deletions/duplications accounted for 56% (42/75) of the segmental aneuploidies (Fig. 4).

**Figure 5.**
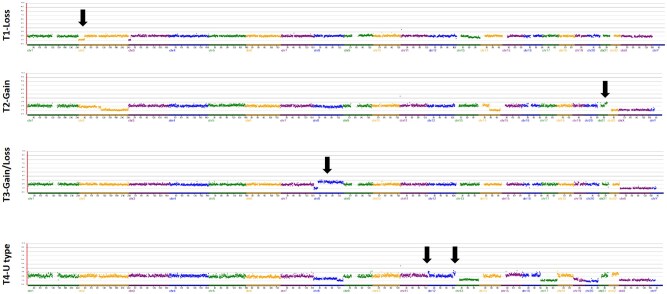
**Types of segmental chromosome abnormalities**. Type 1 (T1): segmental aneuploidy (SA) was observed on chromosome 2, characterized by a segmental deletion (indicated by the black arrow) and on chromosome 3, exhibiting a segmental deletion. Type 2 (T2): segmental duplication was observed on chromosome 21 (indicated by the black arrow), along with segmental deletions on chromosome 2 and 14. Type 3 (T3): segmental duplications at one end of chromosome 8, and a deletion at the opposite end of the same chromosome. Type 4 (T4): segmental duplication was observed at both ends (short arm and long arm) of chromosome 12, with normal copy number variants in the intervening middle section, likely representing a U-type (the shape of the letter ‘U’) configuration.

The 75 breakpoints from 55 embryos were categorized into four types, with a total of 72 breakpoints classified, considering that two breakpoints from three embryos were categorized into the same type. Two common types emerged: segmental duplications (30.6%, 22/72) and segmental deletions (54.2%, 39/72). Additionally, two less common patterns were identified: one involving duplication of one end of the same chromosome with a deletion at the other end (11.1%, 8/72), while the other entails segmental deletion or duplication at both the p-arm and q-arm ends of the chromosome, with the intervening middle section remaining unaffected, likely referred to as U type (4.2%, 3/72) (Fig. 5).

### Relationship between blastocyst quality and euploidy

In the cohort of embryos eligible for biopsy, 41 embryos were analyzed. Among these, 19 blastocysts did not receive a ‘C’ grade in either the inner cell mass or TE, resulting in a euploidy rate of 89.5% (17/19). Meanwhile, 22 blastocysts were graded as ‘C’ for either the inner cell mass or the TE, showing a euploidy rate of 77.2% (17/22). Although a numerical difference of over 10% was observed, this difference was not statistically significant (*P* = 0.53). Specifically, the euploidy rate was 77.8% for blastocysts graded as ‘C’ in the inner cell mass and 76.9% for those graded as ‘C’ in the TE (Supplementary Table S2).

A total of 80 blastocysts were classified as low-quality or arrested blastocysts. All of these embryos received a ‘C’ grading for both the inner cell mass and the TE. The euploidy rate of these embryos was related to the developmental stage of the blastocyst, with rates of 50% for stages IV–V, 42.3% for stage III, and 7.7% for stages I–II (Supplementary Table S2).

## Discussion

Our study presents the largest sample size to date for the detection of chromosome copy number in whole embryos/blastocysts throughout all developmental stages prior to implantation. We identified several novel findings that differ from those typically observed in TE biopsy-based studies.

High-quality blastocysts are very important in the context of IVF treatment. In our study, the euploidy rate of whole blastocysts surpassed 80%, with a mosaic aneuploidy rate as low as 2.5%. Previously, several small-sample studies have performed whole-blastocyst testing on high-quality blastocysts that met biopsy criteria (Kuznyetsov *et al.*, 2018; Huang *et al.*, 2019; Popovic *et al.*, 2019; Navratil *et al.*, 2020; Ou *et al.*, 2020). However, the samples in these studies were often derived from blastocysts found to be chromosomally abnormal (including aneuploid or mosaic aneuploid) following TE biopsy. Consequently, these studies do not contribute directly to comparisons of euploidy rates in whole-blastocyst assessments. Nonetheless, they offer valuable insights into the evaluation of mosaicism diagnosed as aneuploid. For instance, in Ou *et al.*’s study, eight embryos with mosaic aneuploidy rates ranging from 30% to 50% were all confirmed to be euploid upon whole-blastocyst testing (Ou *et al.*, 2020). In contrast, Huang *et al.* (2019) reported several embryos with mosaic aneuploidy rates ranging from 50% to 70%, which were diagnosed as aneuploid through whole-blastocyst testing. However, due to the small sample sizes in these studies, it remains challenging to derive specific mosaic aneuploidy rates following whole-embryo analysis.

In our study, whole blastocysts demonstrated a higher euploidy rate and a lower mosaic aneuploidy rate compared to conventional TE biopsy results. In a large-scale systematic review, which enrolled 7749 blastocysts, only 66% of blastocysts were found to be euploid (Toft *et al.*, 2020). In blastocysts, the mosaic rate of biopsies taken from TE ranges widely, from 2% to 25% (Rana *et al.*, 2023; Popovic *et al.*, 2024). In a large-scale study involving 86 208 blastocysts, a higher mosaic rate of up to 15.8% was reported (Armstrong *et al.*, 2023). Similarly, a multi-center randomized controlled trial reported a mosaic rate of 17% in the analyzed blastocysts (Munné *et al.*, 2019).

The observed increase in the euploid rate and decrease in the mosaic aneuploidy rate may be attributed to the fact that certain embryos, when assessed through TE biopsy, may be diagnosed as mosaic. However, if the entire embryo were examined, it could be classified as euploid. Recent literature has supported this hypothesis. For instance, a study by Wu *et al.* (2021) demonstrated that 50% of embryos initially diagnosed as mosaic were, in fact, euploid following whole-embryo analysis. Similarly, another study involving 2759 mosaic embryos reported a high pregnancy rate of 38% (Treff and Marin, 2021), suggesting that some embryos diagnosed as mosaic based on TE biopsy are actually euploid when the whole embryo is analyzed.

There are three potential explanations for why embryos diagnosed as mosaic through TE biopsy may be classified as euploid when the entire embryo is evaluated. Firstly, low-level mosaicism might be diluted by the higher proportion of normal cells during whole-blastocyst analysis (Ou *et al.*, 2020). Secondly, during blastocyst development, aneuploid cells within different lineages may be selectively depleted through different mechanisms: aneuploid cells in TE exhibit prolonged cell cycle length and senescence, while aneuploid cells in inner cell mass show a higher incidence of apoptosis (Bolton *et al.*, 2016). Notably, previous research has demonstrated that embryos with over 50% euploid cells can be completely rescued, resulting in comparable implantation rates and viability to euploid embryos (Bolton *et al.*, 2016). Thirdly, the original diagnosis of mosaicism in the TE biopsy could potentially be a technical artifact superimposed on euploid profiles. In other words, the embryos may have never been mosaic to begin with.

With a progressive reduction in the proportion of aneuploid cells, the mosaic aneuploidy rate decreased to 2.5% in whole-blastocyst testing, which closely aligns with the mosaicism rate reported in placenta testing (Popovic *et al.*, 2020). Consequently, the euploidy and mosaic rate detected in the biopsiable embryo cohort of our study may more accurately reflect the physiological reality.

It is particularly important to highlight that, in whole blastocyst testing, low-level mosaicism may be diluted by the higher proportion of normal cells. However, the same applies to high-level mosaicism, where the higher proportion of abnormal cells may also mask heterogeneity. Consequently, there remains a possibility of underestimating the presence of mosaicism in whole-embryo analysis.

Few studies have previously focused on low-quality blastocysts before implantation. Embryos cultured *in vitro* are typically evaluated for blastocyst formation on day 5. Those that fail to meet criteria for transfer, freezing or biopsy, are cultured for an additional 24 h, extending to D6. Upon re-evaluation on D6, embryos that still fail to meet the sufficient quality standards for clinical use are generally discarded (Sunkara *et al.*, 2010). However, recent studies suggest that extending culture to Day 7 (D7) may enable a small proportion of otherwise discarded embryos to develop into viable blastocysts (Insogna *et al.*, 2021). Therefore, identifying blastocysts suitable for extended culture to D7 holds significant clinical relevance. We observed that low-quality I–II stage blastocysts exhibit a euploidy rate of 15%, whereas late-D6 blastocysts reaching stage III or above show a euploidy rate of ∼50%. Therefore, in clinical practice, only D6 embryos that progress to stage III or higher should be considered for further culture until D7.

Pre-implantation embryo arrest remains a central focus in assisted reproductive technology research. Since embryos with developmental arrest are relatively easier to obtain, studies on this topic often involve larger sample sizes. A recent study enrolled over 200 arrested embryos/blastocysts, which were categorized based on their developmental stage (cleavage-stage arrest, CP-stage arrest, and blastocyst-stage arrest). The study found that the incidence of aneuploidy in arrested embryos was exceptionally high, reaching 94%, with mitotic aneuploidies affecting multiple chromosomes as the predominant cause (McCoy *et al.*, 2023). This result is consistent with our study, in which we detected an overall aneuploidy rate of 87% in arrested embryos. Furthermore, we analyzed the euploidy rates across different stages of embryo development and observed that the factors contributing to embryo arrest varied according to developmental stage. Mosaic aneuploidy accounted for 90% of the arrests at the cleavage stage, primarily due to chaotic aneuploidy, whereas non-mosaic aneuploidy was the predominant cause of arrest at the blastocyst stage.

In our study, we observed a segmental aneuploidy rate of 4.9% in biopsiable blastocysts. This rate is lower compared to previous studies, where the segmental aneuploidy rate in biopsiable blastocysts ranged from 8.4% (Escribà *et al.*, 2019) to 16% (Babariya *et al.*, 2017). The origin of segmental aneuploidy differs between oocytes and sperm, with rates ranging from 5.7% to 6.3% in oocytes (Hou *et al.*, 2013; Magli *et al.*, 2020), and only 0.4% in sperm (Bell *et al.*, 2020). Interestingly, the segmental aneuploidy rate observed in our biopsiable blastocysts was similar to that of oocytes, but lower than the rate observed in TE biopsy.

Previous studies have shown that 70% of both segmental aneuploidies and mosaic segmental aneuploidies originate from paternal chromosomes (Kubicek *et al.*, 2019). Another study reported that only half of segmental aneuploidies were detected again in re-biopsy samples (Navratil *et al.*, 2020). These results suggest that the majority of segmental aneuploidies likely result from mitotic errors occurring after fertilization (Girardi *et al.*, 2020). As embryos undergo self-correction, segmental aneuploidies originating from mitosis errors may be eliminated. Consequently, the segmental aneuploidy rate observed in entire blastocysts tends to align more closely with the rates observed in oocytes.

Segmental aneuploidy formation may result from S-phase artifacts, which are a potential source of error in WGA. During WGA, the replication domains of single-cell DNA can lead to CN alterations that are misinterpreted as segmental aneuploidies (Van der Aa *et al.*, 2013). Additionally, mosaic segmental aneuploidies may arise from gross structural rearrangements of chromosomes, which could occur as a consequence of replication stress, catenanes, and ultrafine anaphase bridges (Chan *et al.*, 2007; Mizuno *et al.*, 2013; Putnam *et al.*, 2016).

A study showed that breakpoints tend to occur more frequently in gene-poor regions and common fragile sites (Palmerola *et al.*, 2022). In our study, we identified only one high-frequency breakpoint at 1q21, whereas Babariya *et al.* (2017) reported multiple hotspots. This discrepancy may be attributed to the smaller sample size of segmental aneuploidy cases in our study, which included only a few dozen breakpoint data points. In contrast, Babariya’s study included data from nearly 700 breakpoints, enabling a more comprehensive detection of breakpoint regions. Consistent with Babariya’s findings, 1q21 was also identified as a high-frequency breakpoint in their study. The high frequency of breakpoints at 1q21 may potentially be explained by the proximity of the centromere and the lack of genes in this region.

Various types of segmental aneuploidies, including segmental deletions, duplications, and combinations of both deletions and duplications, have been described in the literature based on biopsy data (Tšuiko *et al.*, 2021). In our study, we also observed these three phenotypes. However, a notable issue emerged during our investigation.

Vanneste *et al.* (2009) confirmed that chromosomal instability is a common phenomenon in cleavage-stage embryos. When a chromosome breaks, the break ends become sticky, and these sticky ends may later be stabilized through mechanisms such as telomere addition or centromere inactivation (Guérin and Marcand, 2022). Following chromosomal breaks, one daughter cell may inherit a single terminal deletion, while the other may experience an inverted duplication (Voet *et al.*, 2011). Chromosomes with terminal deletions can be repaired through telomeric healing, leading to a stable segmental deletion/duplication phenotype in subsequent replications. Alternatively, the chromosome may form dicentric chromosomes during replication, which can be stabilized through the inactivation of one centromere. This process results in a stable duplication of one end of the chromosome, accompanied by a deletion at the other end.

Furthermore, we identified a previously unreported pattern of chromosomal breaks, which we have termed the ‘U-type’. This pattern resembles the mechanism of inversion. Typically, a chromosome exhibits a single breakpoint; however, in the U-type chromosomal break pattern, two breakpoints occur on the same chromosome. Following these breaks, the stability of the chromosomal fragments may be maintained through mechanisms such as telomere addition or centromere inactivation.

Our study also has some limitations. First, the sample size, although the largest in human whole-embryo sample analysis to date, remains relatively small after subgroup analysis. Further studies with larger sample sizes are required to validate our findings. Additionally, the limited number of segmental aneuploidy cases in our study constrains our ability to identify additional high-frequency breakpoints. Second, whole-embryo analysis is not a foolproof diagnostic method. In whole blastocyst testing, low-level mosaicism may be diluted by the higher proportion of normal cells; however, the same applies to high-level mosaicism where the higher proportion of abnormal cells may mask heterogeneity. Therefore, there remains a possibility of underestimating the presence of mosaicism. Third, the analysis of the causes behind different types of segmental aneuploidy relies on hypothetical inference and lacks direct experimental validation.

In summary, we investigated the cytogenetic constitution of whole human embryos/blastocysts. Among high-quality blastocysts, the euploidy rate was high, and the mosaic aneuploidy rate was notably low, which suggests that transferring mosaic embryos in the absence of euploid embryos should be reconsidered. Furthermore, if blastocysts reach stage III or above by 6 dpf, nearly half are euploid, supporting embryo culture to D7 in cases where high-quality embryos are lacking. In patient counseling regarding embryo arrest, chaotic aneuploidy emerged as the primary factor contributing to embryonic arrest at the cleavage stage.

## Supplementary Material

hoaf013_Supplementary_Data

## Data Availability

The data that support the findings are available from the corresponding author, upon reasonable request.
