# Supplementary material for: Biopsy vs comprehensive embryo/blastocyst analysis: a closer look at embryonic chromosome evaluation
Source: Hum Reprod Open. 2025 Mar 12;2025(2):hoaf013. doi: 10.1093/hropen/hoaf013 (PMC11928226; doi:10.1093/hropen/hoaf013)
Supplement: hoaf013_Supplementary_Data [file hoaf013_supplementary_data.zip › Supplementary_table_S1.docx]

**Supplementary Table S1: The distribution of embryos among different developmental stages and aneuploidy disputation**

|  |  | Distributaion of aneuploidy | | | | | | | | | |
| --- | --- | --- | --- | --- | --- | --- | --- | --- | --- | --- | --- |
|  |  | Mosaic Aneuploidy | | | |  | Non-Mosaic Aneuploidy | | | | |
|  |  | A | SE | A+SE | Chao |  | A | SE | A+SE | TR | Total |
| **Biopsiable blastocyst** | |  | 1 |  |  |  | 5 | 1 |  |  | 7 |
| **Viable low-quality blastocyst** | |  |  |  |  |  |  |  |  |  | 35 |
|  | I-II stage blastocysts | 2 | 1 | 0 | 5 |  | 3 | 0 | 0 |  |  |
|  | III-V stage blastocysts | 3 | 3 | 2 | 8 |  | 4 | 2 | 2 |  |  |
| **Developmental arrest** | |  |  |  |  |  |  |  |  |  | 97 |
|  | Cleavage stage arrest | 7 | 5 | 4 | 39 |  | 3 | 1 | 0 | 0 |  |
|  | CP stage arrest | 1 | 1 | 2 | 11 |  | 4 | 2 | 0 | 0 |  |
|  | I-II stage blastocysts | 1 | 2 | 2 | 5 |  | 2 | 0 | 0 | 0 |  |
|  | III-V stage blastocysts | 0 | 2 | 0 | 0 |  | 2 | 0 | 1 | 0 |  |
| **Total embryos** | | 14 | 15 | 10 | 68 |  | 23 | 6 | 3 | 0 | 139 |

NR: No Results; EU: Euploidy; AN: Aneuploidy; MO: Mosaic Aneuploidy; NMO: Non-Mosaic Aneuploidy;

A: Whole Chromosomal Aneuploidy; SA: Segmental Aneuploidy; CH: Chaotic Aneuploidy
