# Supplementary material for: Biopsy vs comprehensive embryo/blastocyst analysis: a closer look at embryonic chromosome evaluation
Source: Hum Reprod Open. 2025 Mar 12;2025(2):hoaf013. doi: 10.1093/hropen/hoaf013 (PMC11928226; doi:10.1093/hropen/hoaf013)
Supplement: hoaf013_Supplementary_Data [file hoaf013_supplementary_data.zip › Supplementary-table-S2-post_adjudication_clean_EO.docx]

**Supplementary Table S2: Relationship between blastocyst quality and euploidy**

|  | Stage | Blastocyst quality | Euploidy | Aneuploidy | No Results | Total |
| --- | --- | --- | --- | --- | --- | --- |
| **Biopsiable blastocyst** | | | | | | |
|  | IV-V | | | | | |
|  |  | AA/AB/BA/BB | 17 | 2 |  | 19 |
|  |  | CB/BC | 17 | 5 |  | 22 |
| **Viable low-quality blastocyst and arrested blastocyst** | | | | | | |
|  | IV-V | | | | | |
|  |  | CC | 14 | 14 |  | 28 |
|  | III | | | | | |
|  |  | CC | 11 | 15 |  | 26 |
|  | I-II | | | | | |
|  |  | -- | 2 | 23 | 1 | 26 |
